# Inter-Rater Reliability of Preprocessing EEG Data: Impact of Subjective Artifact Removal on Associative Memory Task ERP Results

**DOI:** 10.3389/fnins.2017.00322

**Published:** 2017-06-16

**Authors:** Steven D. Shirk, Donald G. McLaren, Jessica S. Bloomfield, Alex Powers, Alec Duffy, Meghan B. Mitchell, Ali Ezzati, Brandon A. Ally, Alireza Atri

**Affiliations:** ^1^Mental Illness Research Education Clinical Center, Edith Nourse Rogers Memorial Veterans Hospital (VHA)Bedford, MA, United States; ^2^Department of Neurology, Massachusetts General HospitalBoston, MA, United States; ^3^Biospective, Inc.Montreal, QC, Canada; ^4^Quinnipiac Medical School, Quinnipiac UniversityNorth Haven, CT, United States; ^5^Boston University School of Medicine, Boston UniversityBoston, MA, United States; ^6^New Jersey Medical School, Rutgers UniversityNew Brunswick, NJ, United States; ^7^Department of Mental Health, Tewksbury HospitalTewksbury, MA, United States; ^8^Department of Neurology, Albert Einstein College of MedicineNew York, NY, United States; ^9^Department of Neurosurgery, University of LouisvilleLouisville, KY, United States; ^10^Ray Dolby Brain Health Center and California Pacific Medical Center Research Institute, California Pacific Medical CenterSan Francisco, CA, United States; ^11^Department of Neurology, Center for Brain/Mind Medicine, Brigham and Women's HospitalBoston, MA, United States; ^12^Harvard Medical School, Harvard UniversityBoston, MA, United States

**Keywords:** EEG/ERP, memory, preprocessing, inter-rater reliability, artifacts

## Abstract

The processing of EEG data routinely involves subjective removal of artifacts during a preprocessing stage. Preprocessing inter-rater reliability (IRR) and how differences in preprocessing may affect outcomes of primary event-related potential (ERP) analyses has not been previously assessed. Three raters independently preprocessed EEG data of 16 cognitively healthy adult participants (ages 18–39 years) who performed a memory task. Using intraclass correlations (ICCs), IRR was assessed for Early-frontal, Late-frontal, and Parietal Old/new memory effects contrasts across eight regions of interest (ROIs). IRR was good to excellent for all ROIs; 22 of 26 ICCs were above 0.80. Raters were highly consistent in preprocessing across ROIs, although the frontal pole ROI (ICC range 0.60–0.90) showed less consistency. Old/new parietal effects had highest ICCs with the lowest variability. Rater preprocessing differences did not alter primary ERP results. IRR for EEG preprocessing was good to excellent, and subjective rater-removal of EEG artifacts did not alter primary memory-task ERP results. Findings provide preliminary support for robustness of cognitive/memory task-related ERP results against significant inter-rater preprocessing variability and suggest reliability of EEG to assess cognitive-neurophysiological processes multiple preprocessors are involved.

## Introduction

Event-related potentials (ERPs) continue to be a popular tool in clinical and pharmacological research to assess cognitive-neurophysiological processes. Given its non-invasive nature, high temporal sensitivity, and relative low cost and subject-burden, ERPs may provide an accessible and accurate clinical research biomarker to detect or track changes in cognitive-neurophysiological function or dysfunction due to aging, disease, or drug effects (Cecchi et al., 2015).

Any viable cognitive-neurophysiological or neuropsychological measure must demonstrate measurement reliability, especially if used in clinical studies spanning weekweeks or monthmonths. While stability of ERPs and their EEG recordings can be affected by processes such as sleep deprivation (Murphy et al., 2006; Boonstra et al., 2007) and mood (Cavanagh and Geisler, 2006), ERPs have shown moderate to strong test-retest reliability across a range of cognitive paradigms and their corresponding components (McEvoy et al., 2000; Cassidy et al., 2012).

For the current study, we investigated the inter-rater reliability (IRR) and potential influence of preprocessing by different raters (i.e., different processors) on memory-task ERP results. Processing of EEG data contains a subjective step to remove presumed artifacts, which, even if not explicitly stated in published reports, is presumed to have been performed. Artifacts in the EEG data include effects from eye blinks, high-frequency noise, drift, and “unusually” flat data that may signify faulty electrodes (Tatum et al., 2011). Most critically, the manner by which eye activity is corrected can affect the spatial distribution of the EEG (Berg and Scherg, 1994). During preprocessing, EEG data is typically visually inspected and segments containing residual artifacts are removed (Tatum et al., 2011). The possibility of this subjective component of the processing of EEG/ERP data potentially affecting the outcomes of the subsequent analysis has not been investigated. In this study, we aimed to assess IRR and the robustness of memory-task ERP results to variable rater preprocessing. It is conceivable that preprocessing IRR effects could particularly affect studies that occur at multiple sites or longitudinal studies with staff changes, those studies more likely to involve multiple raters, and/or that involve paradigms with fewer trials due to real-world limitations (e.g., health or comfort of a vulnerable population); factors that are often involved in clinical and pharmacological studies. In this study, we investigate IRR of EEG preprocessing in a paired-associative memory-task ERP paradigm to assess whether possible inter-rater preprocessing effects, and preprocessing in general, will substantially alter the outcome of the primary ERP analyses (i.e., the expected ERP effects of interest).

## Methods

To investigate the IRR of EEG preprocessing, three raters preprocessed all raw EEG data independently from a study investigating face-name memory in a fully crossed IRR design (see Mitchell et al., 2016). Intraclass Correlation Coefficients (ICCs) were calculated to measure IRR for each of three *a priori* defined ERP effects. Analysis of the face-name paradigm effect was also conducted for each rater separately to determine if individual rater preprocessing may have had an effect on interpretation of results. Unprocessed EEG data was also analyzed to further investigate the impact of preprocessing on ERP effects.

### Participants

Each preprocessor (i.e., rater) had a Bachelor's degree. One rater had considerable training (by one of the lab investigators/authors, AE) and experience with EEG methodology and preprocessing while the other two were novices. The two novice raters received ~20 h of orientation and training on EEG methodology and preprocessing from the experienced rater. In addition to subjective impact between processors, the level of training would also inform us with regard to the amount training needed, and provided insight into the generalizability of the results. All raters followed the same steps independently which were available for reference within a lab manual.

Study participants consisted of 16 healthy adults (ages 18–39) who underwent neuropsychological battery, followed by completing a face-name memory paradigm with simultaneous EEG recording and eye-tracking. Participants were native English speakers, and they had corrected 20/30 or better color vision. Participants were required to have no history of any neurologic or psychiatric conditions and could not be taking psychoactive medications. All study participants provided written informed consent before participating and were paid 70 USD for their participation. This project was approved by the Bedford Department of Veteran's Affairs Hospital Institutional Review Board.

### Procedure

#### Face-name recognition memory paradigm

The Face-Name paradigm consists of a study phase, during which participants viewed 40 different face-name pairs; of which 20 were repeated 4 times (4R) and 20 were only presented once (1R). Following the study phase, there was a test phase, during which participants were presented with 80 face-name pairs [20 1R (old), 20 4R (old), and 40 N (new)] and asked to indicate if each face-name pair was “new” or “old.” We *a priori* defined three ERP effects of interest that occur across the 2-seconds interval during which a participant is determining if a stimulus is “old” or “new.” The three effects are: (1) the “early frontal effect” or “FN400,” observed in recognition memory paradigms at bilateral frontal electrode sites during the 300–500 ms interval, is associated with enhanced familiarity (Curran, 2000; Curran and Cleary, 2003; Curran and Hancock, 2007; Rugg and Curran, 2007); (2) the “parietal old/new effect,” observed at parietal electrode sites, generally with left greater than right activation during the 500–800 ms interval, is associated with recollection (Herron et al., 2003; Vilberg and Rugg, 2009); and (3) the “late frontal effect,” found bilaterally at frontal electrode sites, typically with greater right activation, during the 1,000–1,800 ms interval, is associated with post-retrieval verification and monitoring processes (Ally and Budson, 2007) or with a more generic form of self-monitoring (Hayama et al., 2008). See Mitchell et al. (2016) for more detail regarding the task paradigm.

### EEG data acquisition

**Figure 1:**
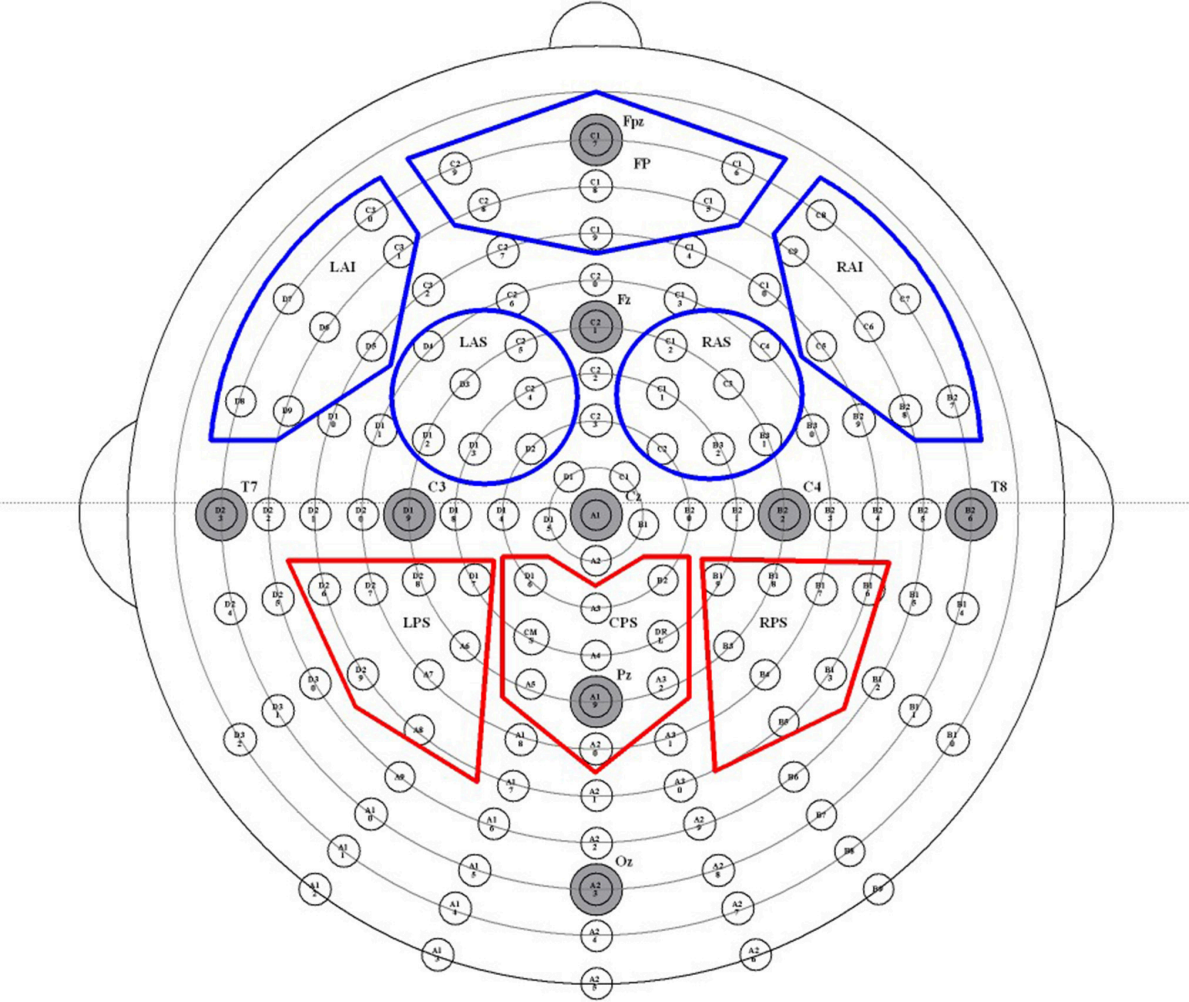
Map of high-density EEG electrode locations and their corresponding regions of interest (ROIs). Electrodes are divided into 10 ROIs, abbreviated as follows: left anterior inferior (LAI), frontal pole (FP), right anterior inferior (RAI), left anterior superior (LAS), right anterior superior (RAS), left posterior superior (LPS), posterior medial (PM), right posterior superior (RPS), left posterior inferior (LPI), and right posterior inferior (RPI). The five frontal ROIs are outlined in red, and the three parietal ROIs are outlined in blue.

As described in Mitchell et al. (2016), an Active Two-electrode cap (Behavioral Brain Sciences Center, Birmingham, UK) was fastened below the chin of the participants. One hundred and twenty-eight Ag-AgCl BioSemi (Amsterdam, the Netherlands) “active” electrodes were then connected to the cap in a configuration that places each electrode in equidistant concentric circles from 10 to 20 position, Cz. See Figure 1 for a visual representation of electrode placement. In addition, mini-biopotential electrodes were placed behind each ear on each mastoid process. Below the left eye and on the outer canthus of each eye, bipolar electrodes were placed to record vertical and horizontal EOG activity. A small amount of a conductive gel was applied to each electrode, and the electrodes were connected to the machine that records EEG brain waves. EEG data was acquired using 128-channels and recorded continuously during each design phase.

### EEG data processing and statistical analysis

**Figure 2:**
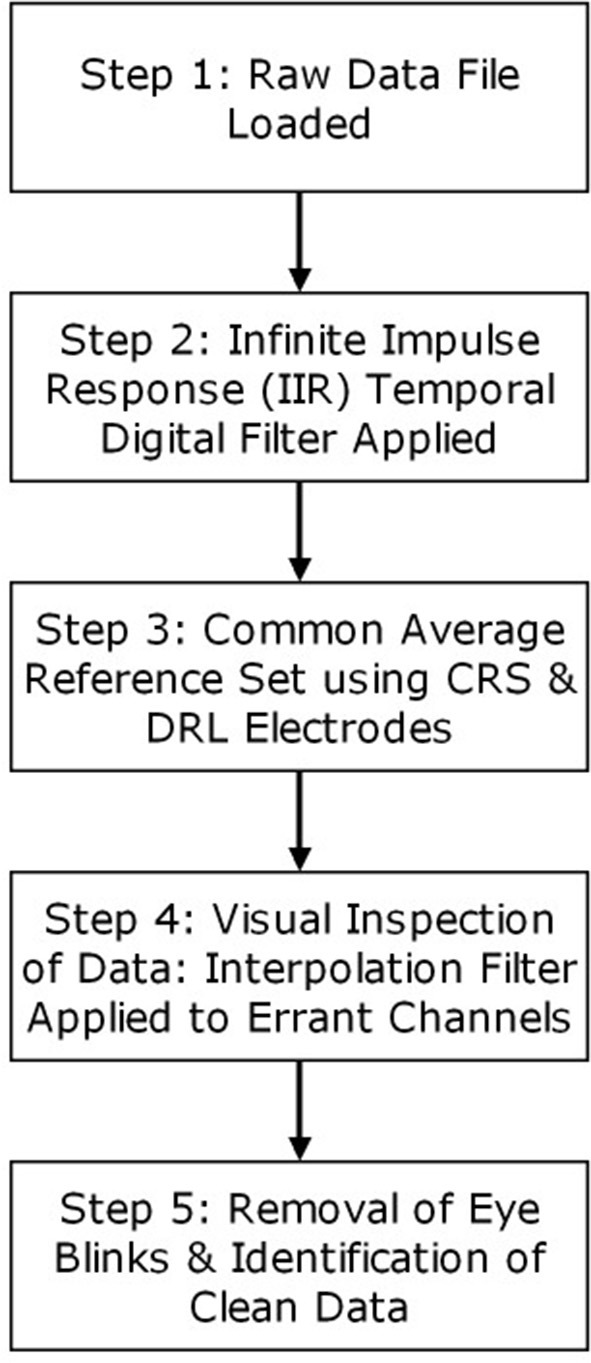
General steps involved in the processing of raw EEG data for each processor.

Each rater/processor processed all data independently and used EMSE Suite software (Cortech Solutions, Wilmington, NC, USA) to perform the following steps in accordance with detailed written instructions. A digital IIR (infinite impulse response) bandpass filter from 0.03 to 30 Hz (−6 db/octave; zero-phase/two-pass Butterworth) was applied to the continuous data. The common average reference (AVE) was employed using CRS and DRL. All channels were then referenced to this common average. All channels were visually inspected for unusually flat data, high-frequency noise, drift, and relative consistency with neighboring channels. Channels identified as outliers were spatially interpolated using spherical splines (Perrin et al., 1989). A maximum of four channels per quadrant were filtered in this way. In the instances where all channels were aberrant for a section of time, those sections were entirely removed from the analysis. Ocular artifacts were corrected using a variant of spatial principal component analysis (PCA) designed to protect against over-correction in frontal regions. Processors identified representative segments of clean (artifact free) data and also representative segments containing ocular artifacts. PCA was performed on the artifact-to-clean spatial contrast matrix (i.e., artifact covariance matrix after pre- and post-multiplication by the inverse symmetric square root of the clean data covariance matrix). The processor inspected the resulting scree plot (on a logarithmic scale) to select a small number (≤ 5) of artifact components to remove, after which the ocular correction matrix was applied to the data (Pflieger, 2001). Figure 2 provides a flowchart of the general steps taken by each processor. Average ERPs were constructed from trials containing 2,000 ms epochs of raw data, of which the first 200 ms was a pre-stimulus period used to baseline-correct the following 1,800 ms period. ERP data was summarized by averaging activity across channels in eight regions of interest (ROIs), each of which consisted of seven or eight channels.

To assess IRR, mixed, absolute agreement average-measures ICCs (McGraw and Wong, 1996; Hallgren, 2012) were calculated for each of the three time intervals of interest and their corresponding ROIs for each effect of interest. ICCs provided a quantitative measure of absolute agreement between the three processors who independently preprocess the participants' EEG data. ICCs range from 0 to 1, where values <0.40 are considered poor, values from 0.40 to 0.59 are considered fair, values 0.60 to 0.74 are considered good, and values 0.75 and higher are considered excellent (Cicchetti, 1994).

To compare ERP brain activity across different conditions for each processor/rater, we conducted three separate multivariate repeated measures analysis on each rater's data for each of the three time intervals of interest and their corresponding ROIs in order to assess the “early frontal effect” (300–500 ms at the five frontal ROIs), the “parietal old/new effect” (500–800 ms at the three parietal ROIs), and the “late frontal effect” (1,000–1,800 ms at the five frontal ROIs). ERP activity was averaged across the time period of interest for all correct responses by stimulus type. “Hits” for 1R and 4R face-name pairs, and correct rejections for N face-name pairs were considered correct responses.

## Results

**Figure 3:**
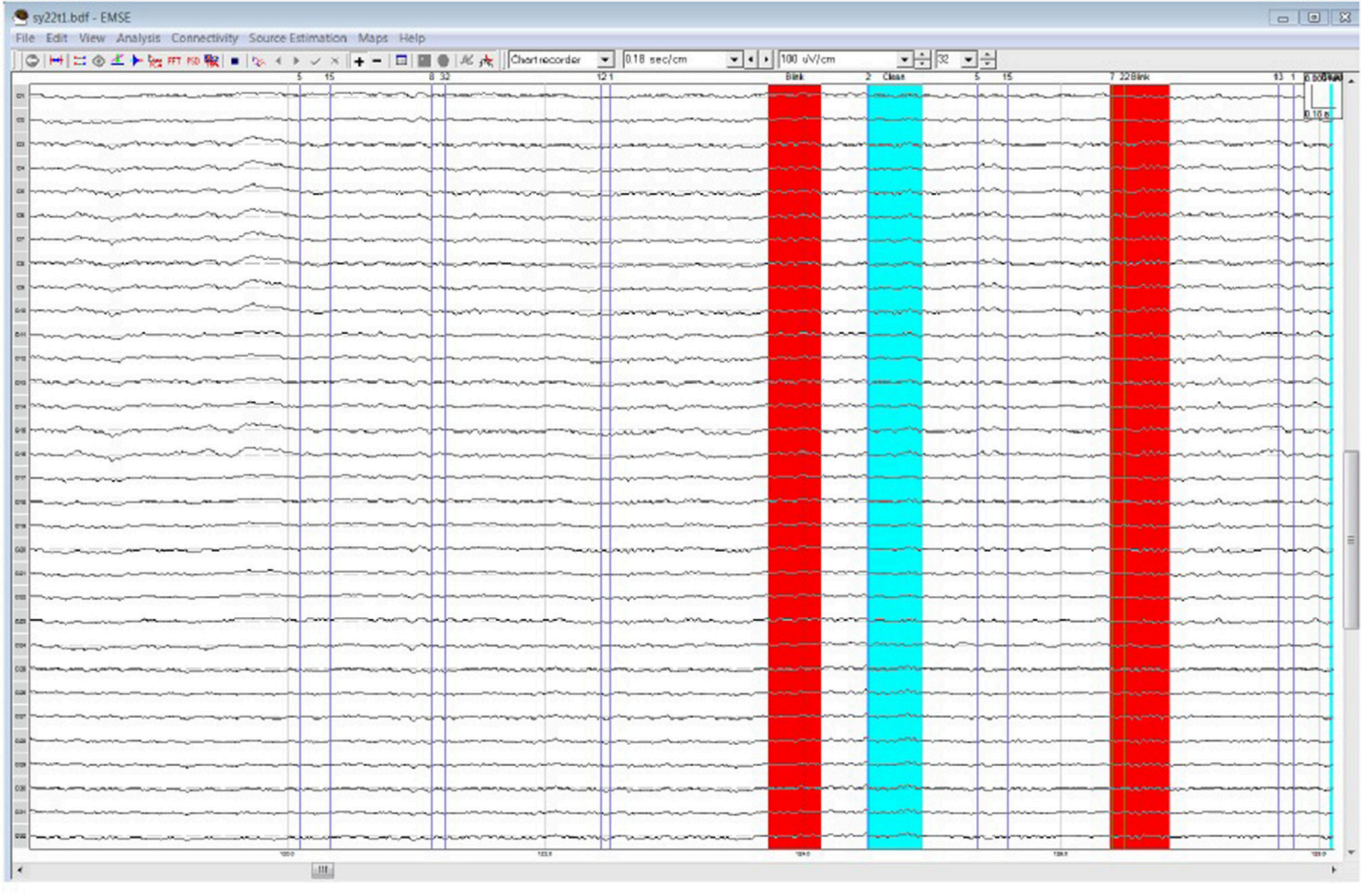
Processor A's selection of clean data (cyan) and blinks (red) within the frontal quadrant of the brain between 119 and 128 ms.

**Figure 4:**
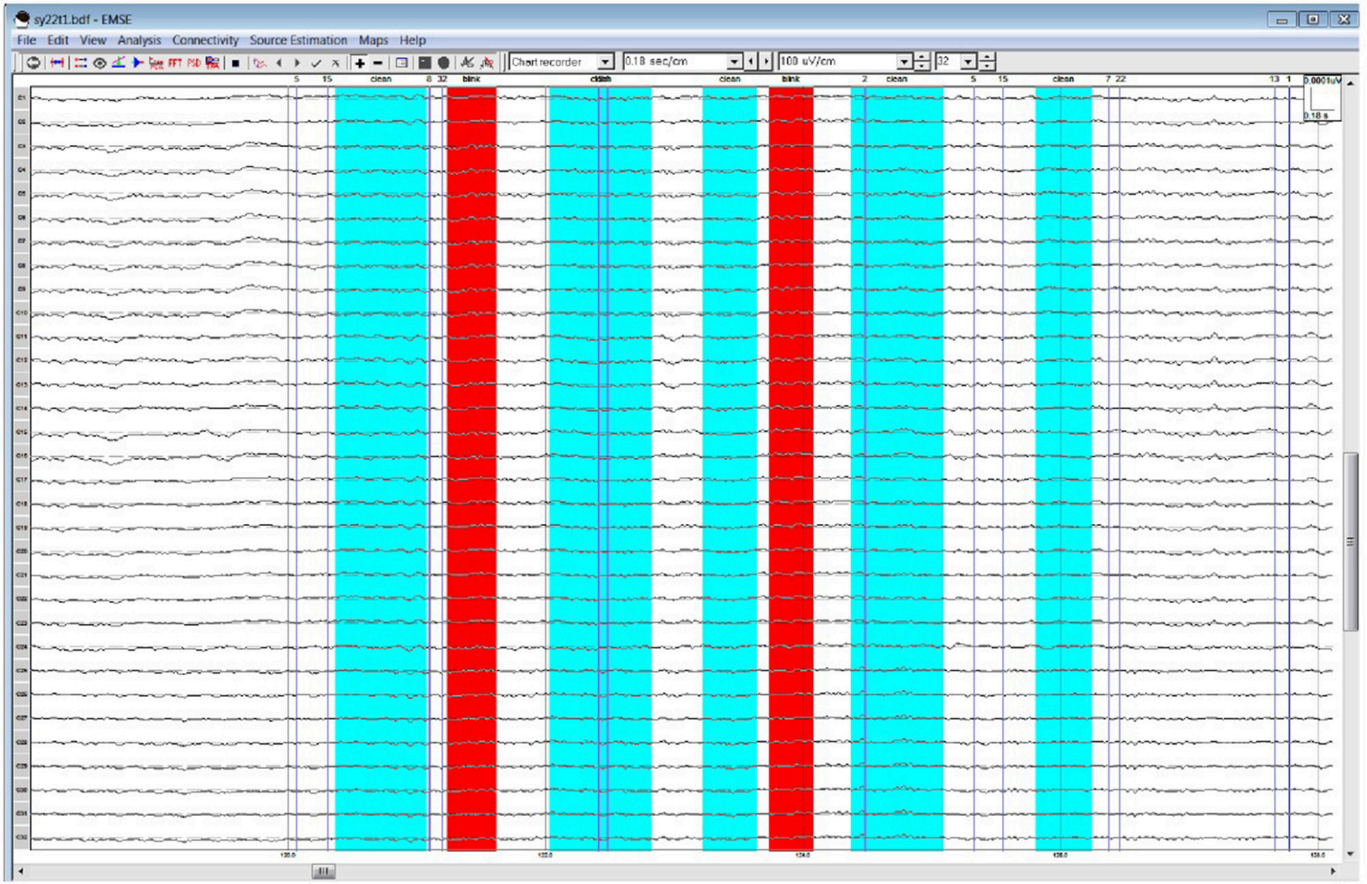
Processor B's selection of clean data (cyan) and blinks (red)·within the frontal quadrant of the brain between 119 and 128 ms.

**Figure 5:**
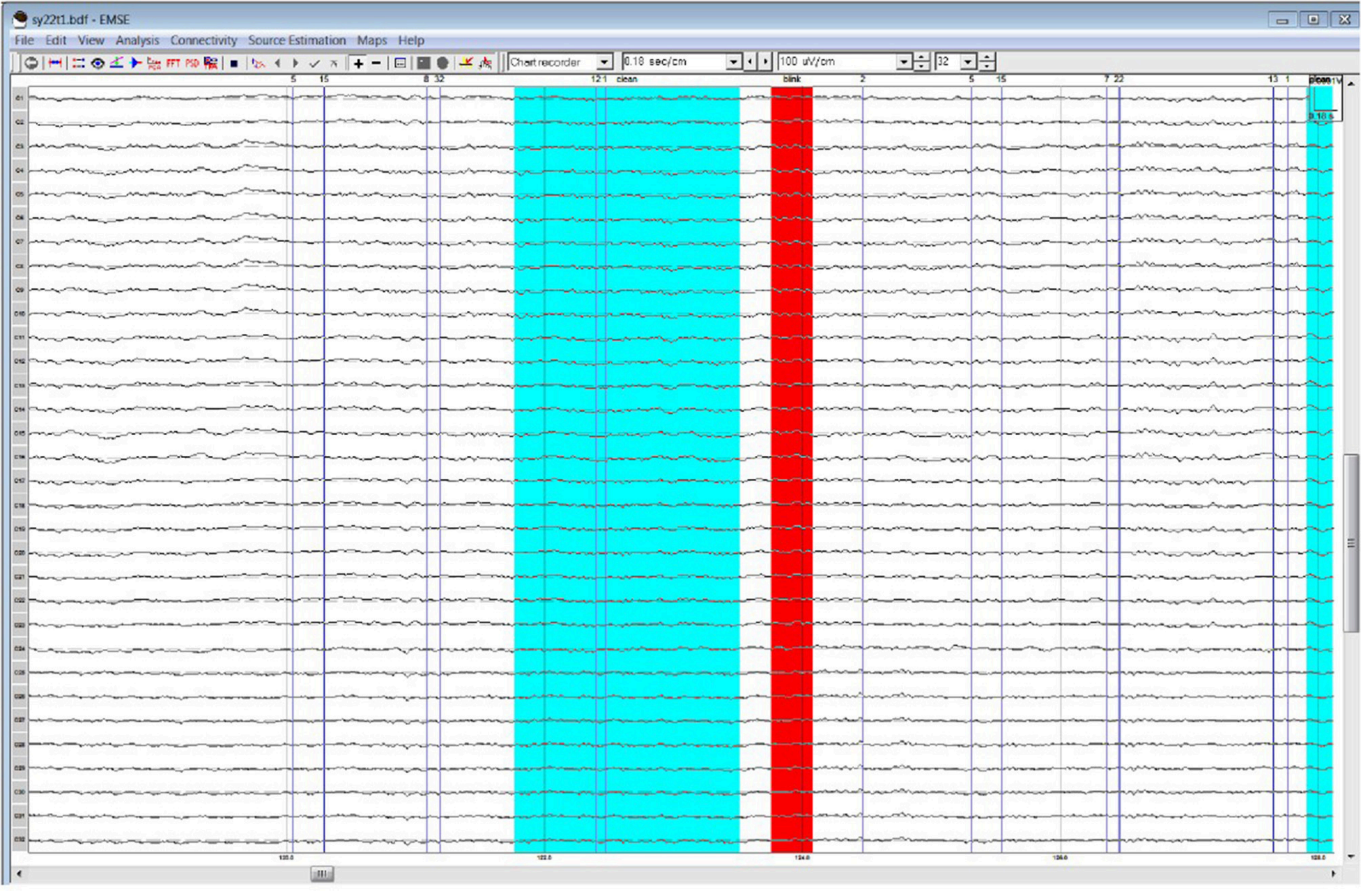
Processor C's selection of clean data (cyan) and blinks (red) within the frontal quadrant of the brain between 119 and 128 ms.

Table 1 lists all ICCs for each area/epoch of interest. Only 2 of 26 ROI ICCs were below 0.75. The two ICCs occurred in the Frontal Pole (FP) and were specific to the 4R-N early-frontal contrast, ICC = 0.63, and the 1R-N late-frontal contrast, ICC = 0.60. Although much lower than other areas, the ICCs are considered good (Cicchetti, 1994). In non-FP regions, the IRR was excellent. Early-frontal effects for 1R-N contrasts and parietal effects of interest had very high ICCs (*Median* = 0.92, *Range* = 0.75–0.98). In addition, Figures 3–5 provide a visual example demonstrating the differences in processing between the three processors. When contrasting the three samples, it becomes clear that although there is some overlap (e.g., the selection of an eye blink at 124 ms for all three processors), the selection of clean data and eye blinks vary and are idiosyncratic to each processor.

**Table 1:** Intraclass correlation coefficients for regions and epochs of interest by condition.

	**ICC**
Early Frontal LAS 4R-N	0.91
Early Frontal FP 4R-N	0.63
Early Frontal RAS 4R-N	0.94
Early Frontal LAI 4R-N	0.86
Early Frontal RAI 4R-N	0.75
Early Frontal LAS 1R-N	0.96
Early Frontal FP 1R-N	0.90
Early Frontal RAS 1R-N	0.98
Early Frontal LAI 1R-N	0.97
Early Frontal RAI 1R-N	0.89
Parietal LPS 4R-1R	0.96
Parietal PM 4R-1R	0.98
Parietal RPS 4R-1R	0.94
Parietal LPS 4R-N	0.97
Parietal RPS 4R-N	0.96
Parietal PM 4R-N	0.87
Late Frontal LAS 4R-N	0.88
Late Frontal FP 4R-N	0.89
Late Frontal RAS 4R-N	0.95
Late Frontal LAI 4R-N	0.86
Late Frontal RAI 4R-N	0.88
Late Frontal LAS 1R-N	0.90
Late Frontal FP 1R-N	0.60
Late Frontal RAS 1R-N	0.94
Late Frontal LAI 1R-N	0.93
Late Frontal RAI 1R-N	0.78

*ERP, event-related potential; LAS, left anterior superior; FP, frontal pole; RAS, right anterior superior; LPS, left posterior superior; RPS, right posterior superior; PM, posterior medial; RAI, right anterior inferior; RAS, right anterior superior; ICC, intraclass correlation coefficient; 4R, 4 times repeated; 1R, 1 time repeated; N, Novel. Early Frontal Effect = 300–500 ms; Parietal Effect = 500–800 ms; Late Frontal Effect = 1,000–1,800 ms*.

*ERP effects of interest were calculated by creating difference scores between conditions indicated for a given region of interest and a given time interval (e.g., Early Frontal Effect FP 1R > N indicates a difference score between averaged microvolts for 1R FN pairs vs. Novel FN pairs in the frontal pole region of interest during the 300–500 ms time interval)*.

**Figure 6:**
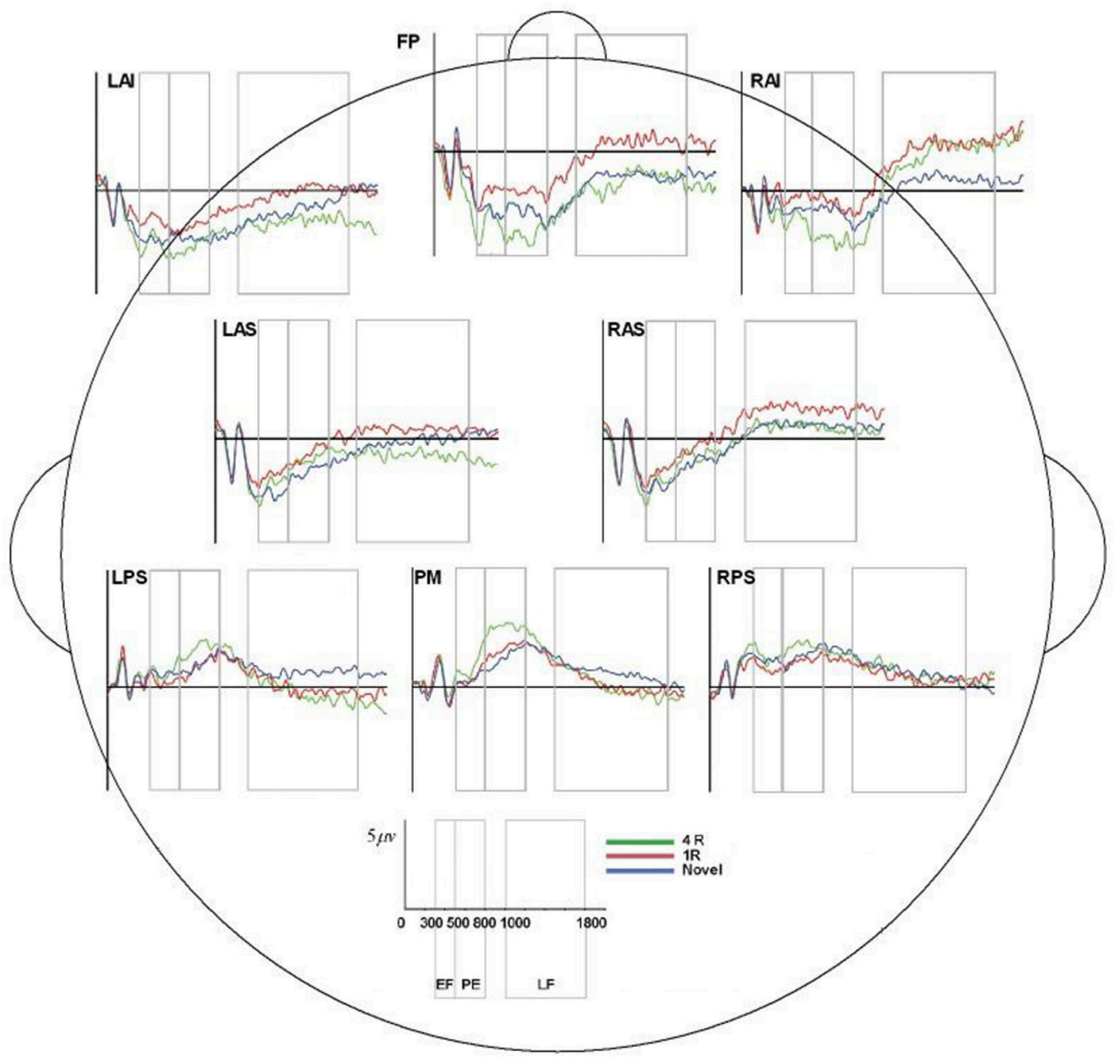
ERP, event-related potential; LAS, left anterior superior; FP, frontal pole; RAS, right anterior superior; LPS, left posterior superior; RPS, right posterior superior; PM, posterior medial; RAI, right anterior inferior; RAS, right anterior superior; 4R, presented 4 times; 1R, presented 1 time; N, Novel; EF, Early Frontal Effect, 300–500 ms; PE, Parietal Effect; 500–800 ms; LF, Late Frontal Effect; 1,000–1,800 ms. Average ERP wave forms for each region of interest (ROI) across the three conditions for processor A. *X*-axis represents time (in milliseconds) from 0 to 2,000, and *Y*-axis represents microvolts. Blue lines represent novel face-name (FN) pairs (correct rejections), red lines represent 1-time repeated (lR) FN pairs (hits), and green lines represent 4-times repeated (4R) FN pairs (hits).

**Figure 7:**
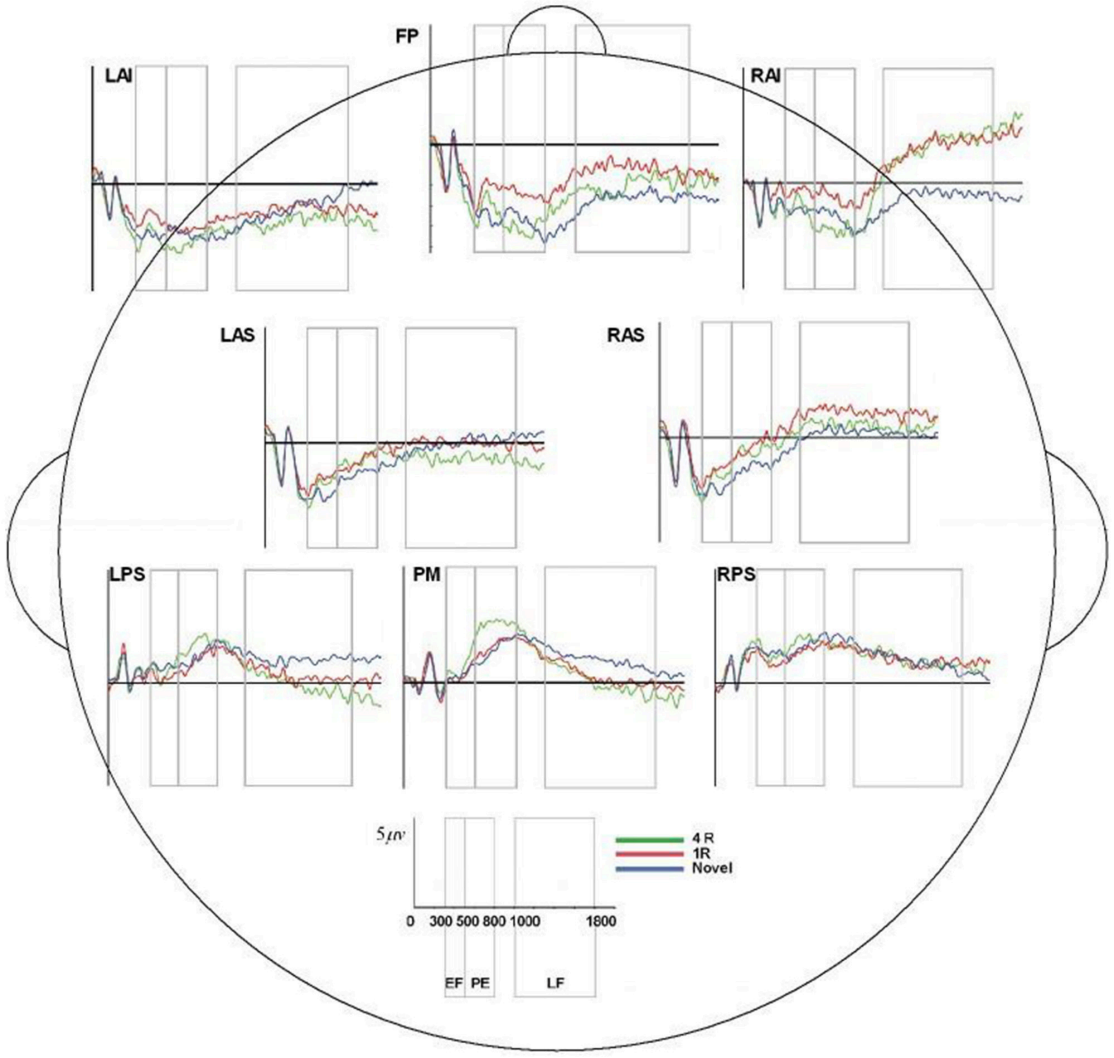
ERP, event-related potential; LAS, left anterior superior; FP, frontal pole; RAS, right anterior superior; LPS, left posterior superior; RPS, right posterior superior; PM, posterior medial; RAI, right anterior inferior; RAS, right anterior superior; 4R, presented 4 times; 1R, presented 1 time; N, Novel; EF, Early Frontal Effect, 300–500 ms; PE, Parietal Effect; 500–800 ms; LF, Late Frontal Effect; 1,000–1,800 ms. Average ERP wave forms for each region of interest (ROI) across the three conditions for processor B. *X*-axis represents time (in milliseconds) from 0 to 2,000, and *Y*-axis represents microvolts. Blue lines represent novel face-name (FN) pairs (correct rejections), red lines represent 1-time repeated (lR) FN pairs (hits), and green lines represent 4-times repeated (4R) FN pairs (hits).

**Figure 8:**
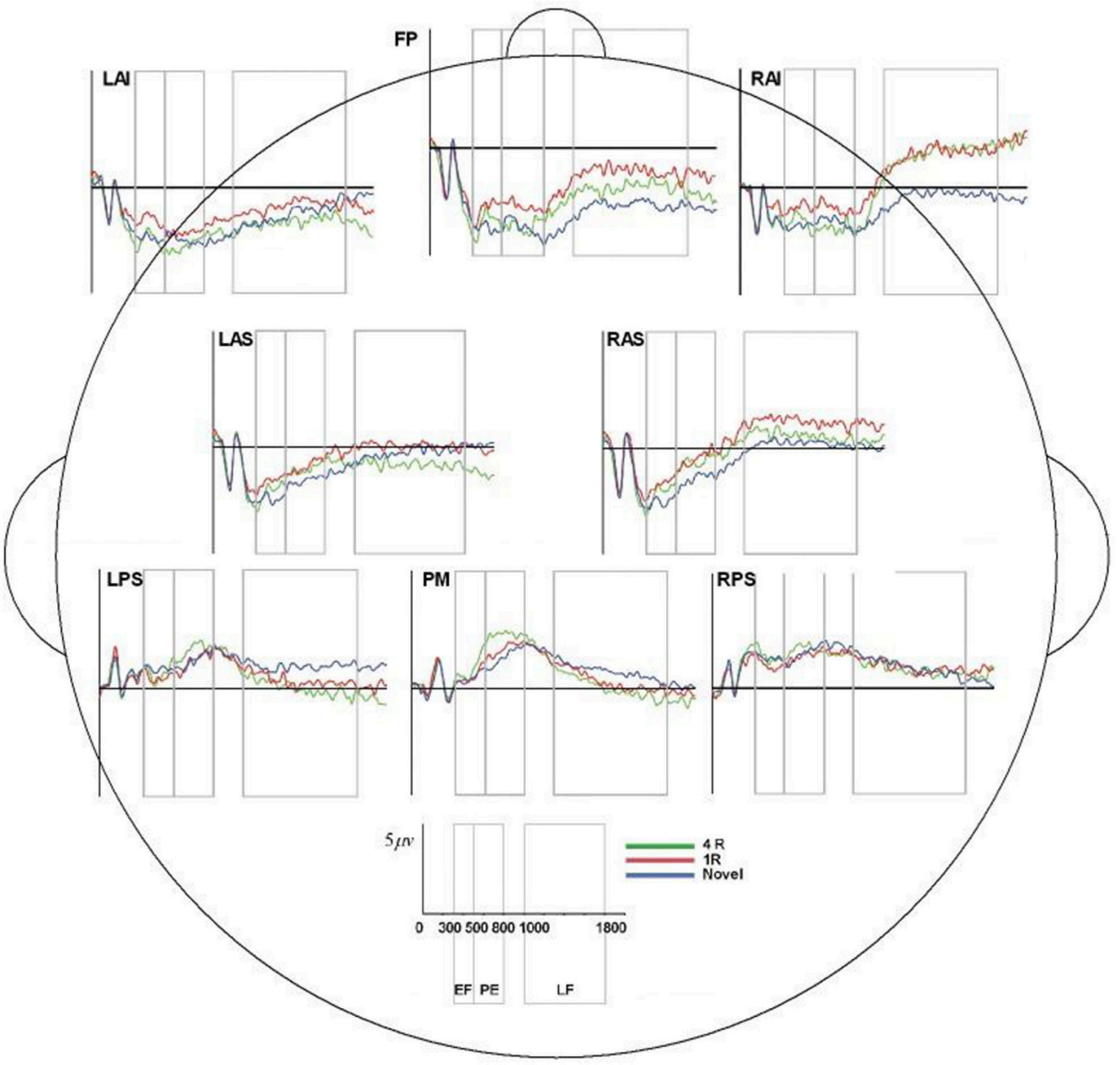
ERP, event-related potential; LAS, left anterior superior; FP, frontal pole; RAS, right anterior superior; LPS, left posterior superior; RPS, right posterior superior; PM, posterior medial; RAI, right anterior inferior; RAS, right anterior superior; 4R, presented 4 times; 1R, presented 1 time; N, Novel; EF, Early Frontal Effect, 300–500 ms; PE, Parietal Effect; 500–800 ms; LF, Late Frontal Effect; 1,000–1,800 ms. Average ERP wave forms for each region of interest (ROI) across the three conditions for processor C. *X*-axis represents time (in milliseconds) from 0 to 2,000, and *Y*-axis represents microvolts. Blue lines represent novel face-name (FN) pairs (correct rejections), red lines represent 1-time repeated (lR) FN pairs (hits), and green lines represent 4-times repeated (4R) FN pairs (hits).

Though the IRR results (ICCs) for area/epoch of interest were strong suggesting that the primary ERP effects would be evident for each processor's data, we also conducted multivariate repeated measures analysis on each rater's EEG preprocessed data to confirm this expectation. All raters produced significant effect of condition and the expected Early Frontal, Parietal, and Late Frontal contrast effects. Finally, analysis of the unprocessed EEG data reproduced the expected Parietal and Late Frontal ERP effects but did not reproduce the Early Frontal effects. Results by rater/processor and unprocessed data are provided in Table 2 and grand average waves by condition for each processor is provided in Figures 6–8.

**Table 2:** Primary ERP effects of interest by processor/rater.

**Processor**	**ERP effect**	***F***
1	Early frontal effect	
	*1R-N contrast*	8.84^*^
	Parietal effect	
	*4R-N contrast*	7.75^**^
	*4R-1R contrast*	11.29^**^
	Late frontal effect	
	*1R-N contrast*	6.64^*^
2	Early frontal effect	
	*1R-N contrast*	10.48^*^
	Parietal effect	
	*4R-N contrast*	11.10^**^
	*4R-1R Contrast*	18.75^***^
	Late frontal effect	
	*1R-N contrast*	7.08^*^
3	Early frontal effect	
	*1R-N contrast*	5.91^*^
	Parietal effect	
	*4R-N contrast*	18.25^***^
	*4R-1R contrast*	22.14^***^
	Late frontal effect	
	*1R-N contrast*	10.37^**^
*Unprocessed*	Early frontal effect	
	*1R-N contrast*	0.05
	Parietal effect	
	*4R-N contrast*	5.10^*^
	*4R-1R contrast*	7.39^**^
	Late frontal effect	
	*1R-N contrast*	6.26^*^

* *p < 0.05*,

** *p < 0.01*,

*** *p < 0.001*.

*ERP, event-related potential; 4R, presented 4 times; 1R, presented 1 time; N, Novel. Early Frontal Effect = 300–500 ms; Parietal Effect = 500–800 ms; Late Frontal Effect = 1,000–1,800 ms*.

*ERP effects of interest were calculated by creating difference scores between conditions indicated for a given region of interest and a given time interval (e.g., Early Frontal Effect FP 1R > N indicates a difference score between averaged microvolts for 1R FN pairs vs. Novel FN pairs in the frontal pole region of interest during the 300–500 ms time interval)*.

## Discussion

In this study, we found EEG preprocessing IRR to be high, and that preprocessing by different raters did not significantly affect results of the primary analyses of interest (i.e., expected ERP memory effects of interest). With the exception of the Frontal Pole, which had good to excellent ICCs, other regions produced excellent IRR, with the vast majority of ICCs in the excellent range of >0.75. In addition, visual examples were provided to demonstrate how the preprocessing of the data varied across processors; yet, despite these differences the effect remained robust and did not undermine the ERP effects. To our knowledge, preprocessing IRR and its possible effects due to subjective removal of artifacts during the preprocessing of EEG data had not been previously reported. Finally, we observed that un-preprocessed EEG data did not fully reproduce results of the primary memory-task effects, which further supports the value of preprocessing of ERP data for the detection of the effect of interest.

That the Frontal Pole showed the least consistency across raters may not be surprising. This region is most susceptible to common artifacts such as facial movement and eye blinks, and thus can yield “noisier” data. The Early-frontal/FN400 effect was also not observed in the primary analysis of the raw EEG data, which suggests that memory-task ERP data may be noisier in this region and supports that preprocessing may be of particular value for signal detection sensitivity in frontal regions. In contrast, the parietal lobes, a region far from these common nuisance artifacts, reflected a relative island of stability; IRR was high in parietal regions and parietal old/new memory effects were observed with ease even from the analysis of the unpreprocessed data. Current findings could potentially inform future study designs that focus on particular cognitive processes or when significant amounts of facial or eye movements are expected. For example, processes that involve the prefrontal cortex or studies which involve a population susceptible to movements (e.g., Parkinson's patients) will surely benefit from preprocessing, but the specific preprocessor should have little effect on the outcome.

Despite observing relatively lower IRR within the late frontal epoch, the Late-Frontal effect itself remained robust, and was even observed in the primary analysis of unpreprocessed data. We posit that this is likely due to longer activity and, consequently, longer time interval of neural activity being measured: the late frontal epoch (1,000–1,800 ms) is four times as long as the early frontal epoch (300–500 ms). The longer activity interval for the late frontal epoch would provide more time to capture a signal and could potentially provide an increase in signal to noise ratio.

Although our ERP paradigm was one of paired-associate memory, the findings are the first to demonstrate the robustness of EEG data to potential inter-rater preprocessing variability and its lack of substantial influence upon memory-task ERP effects. This robustness of EEG data to inter-rater preprocessing effects may translate to other cognitive paradigms that produce or heavily engage similar networks, ROIs, and effects (particularly memory-related parietal effects and executive/frontal effects), especially when considering the observed test-retest reliability across cognitive paradigms and their corresponding components (Cassidy et al., 2012). The most salient observations regarding ICCs that may generalize well to similar ERP cognitive paradigms include very robust parietal old/new (“recollection-based”) ERP effects and Early-frontal/FN400 effects for 1R-N contrasts (“familiarity-based”) which had extremely high mean ICCs (0.94–0.95). We posit that ICCs would be even higher in simpler cognitive ERP paradigms that produce better signal to noise ratio characteristics such as stimulus discrimination, attentional, and sensorimotor paradigms. We also speculate that conditions or characteristics that may more greatly affect the frontal ERP signal to noise ratio, such as error-related negativity for unaware compared to uncertain responses and aware errors (Navarro-Cebrian et al., 2013) and sex (Bourisly and Pothen, 2016) would produce impacts on ICCs. Lastly, these results suggest that with only minimal training and the availability of a manual, novice preprocessors can produce reliable results.

Although, we did find robust signal across all raters and successfully demonstrated the limited effects of rater subjectivity had upon finding the expected ERP effects, we do not wish to minimalize the importance of other factors involved in the processing of raw EEG data, including the filters employed, number of channels interpolated, and the method of correcting ocular artifacts. The choice of a reference is also critical in this regard. There is no universal reference scheme (Kayser and Tenke, 2010; Nunez, 2010). Although, it has been previously demonstrated that the use of different references can have substantial impact on the outcomes of EEG and ERP findings (e.g., Yao, 2001), the choice of reference is often based upon the nature of the research, the number of channels used, the brain networks of interest, or sometimes, established practice. Recently, systematic comparisons of different references have been made (Chella et al., 2016; Lei and Liao, 2017). For example, Lei and Liao (2017) demonstrated the infinity reference obtained by the reference electrode standardization technique (REST) appeared to have the least amount of relative error. Similar to the evolving directives of reference employment, we too hope to provide insight into best practices of performing EEG/ERP research by demonstrating the influence processors may have on the final outcome of an EEG/ERP study.

Study limitations include that it was performed at one site, using one system, and involved cognitively normal subjects; all characteristics that would be expected to produce relatively higher ERP signals with lower variability, as compared to, for example, impaired subjects tested at different sites using different EEG acquisition and analysis platforms. Current results, though promising, may not generalize for older individuals or those with cognitive impairments, psychiatric conditions or brain injury—preprocessing IRR and ERP effects should be assessed further in these populations. Although the results observed with this ERP paradigm could translate to other ERP paradigms that involve similar cognitive processes and brain regions, future studies should assess IRR results for different cognitive paradigms and across different populations. Finally, these results lend further support for the value of “subjective artifact removal” (aka. rater-dependent preprocessing of EEG data) to achieve higher sensitivity to detect ERP-related memory-effects of interest, particularly the Early-frontal/FN400 effect.

With interest in cognitive-neurophysiological outcome measures that correlate with synaptic networks in multi-site studies and clinical trials, there is a need to further assess potential sources of extraneous variability that may affect ERP results. Findings from this study support the robustness of ERP results to inter-rater preprocessing differences and suggest viability of ERP assessments performed by multiple processors; a likely by-product of multi-site ERP studies.

## Ethics statement

This study was carried out in accordance with the recommendations of “Bedford Hospital Research Guidelines, and reviewed by the Hospital's Institutional Review Board” with written informed consent from all subjects. All subjects gave written informed consent in accordance with the Declaration of Helsinki. The protocol was approved by the Edith Nourse Rogers Memorial VA Hospital IRB.

## Author contributions

SS: Involved in the study development, analysis of data, and writing of the manuscript. DM: Involved in the study development, analysis of data, and writing of the manuscript. JB: Involved in the analysis of data and writing of the manuscript. AP: Involved in the analysis of data and writing of the manuscript. AD: Involved in the analysis of data and writing of the manuscript. MM: Involved in the study development, analysis of data, and writing of the manuscript. AE: Involved in the analysis of data and writing of the manuscript. BA: Involved in the study development and writing of the manuscript. AA: Involved in the study development, analysis of data, and writing of the manuscript.

### Conflict of interest statement

The authors declare that the research was conducted in the absence of any commercial or financial relationships that could be construed as a potential conflict of interest.
